# Single-cell transcriptomics reveals the cell fate transitions of human dopaminergic progenitors derived from hESCs

**DOI:** 10.1186/s13287-022-03104-7

**Published:** 2022-08-13

**Authors:** Lingmin Liang, Yao Tian, Lin Feng, Chaoqun Wang, Guihai Feng, Glyn Nigel Stacey, Ng Shyh-Chang, Jun Wu, Baoyang Hu, Wei Li, Jie Hao, Liu Wang, Yukai Wang

**Affiliations:** 1 State Key Laboratory of Stem Cell and Reproductive Biology, Institute of Zoology, Chinese Academy of Sciences, Beijing, 100101 China; 2 Savaid Medical School, University of Chinese Academy of Sciences, Beijing, 100049 China; 3 Institute for Stem Cell and Regeneration, Chinese Academy of Sciences, Beijing, 100101 China; 4 Beijing Institute for Stem Cell and Regenerative Medicine, Beijing, China; 5 National Stem Cell Resource Center, Chinese Academy of Sciences, Beijing, 100101 China; 6 University of Chinese Academy of Sciences, Beijing, 100864 China; 7 International Stem Cell Banking Initiative, Hertfordshire, UK

**Keywords:** Dopaminergic (DA) neurons, Pluripotent stem cells (PSCs), Single-cell RNA sequencing (scRNA-seq), Cell therapy, Parkinson’s disease (PD)

## Abstract

**Background:**

Midbrain dopaminergic (DA) progenitors derived from human pluripotent stem cells are considered to be a promising treatment for Parkinson’s disease (PD). However, the differentiation process produces undesired cell types, which influence the in vivo evaluation of DA cells. In this paper, we analyze the cell fate choice during differentiation and provide valuable information on cell preparation.

**Methods:**

Human embryonic stem cells were differentiated into DA progenitors. We applied single-cell RNA sequencing (scRNA-seq) of the differentiation cells at different time points and investigated the gene expression profiles. Based on the differentially expressed genes between DA and non-DA cells, we investigated the impact of *LGI1* (DA enriched) overexpression on DA differentiation and the enrichment effect of CD99 (non-DA enriched) sorting.

**Results:**

Transcriptome analyses revealed the DA differentiation trajectory as well as non-DA populations and three key lineage branch points. Using genetic gain- and loss-of-function approaches, we found that overexpression of *LGI1*, which is specific to EN1^+^ early DA progenitors, can promote the generation of TH^+^ neurons. We also found that choroid plexus epithelial cells and DA progenitors are major components of the final product (day 25), and CD99 was a specific surface marker of choroid plexus epithelial cells. Sorting of CD99^−^ cells eliminated major contaminant cells and improved the purity of DA progenitors.

**Conclusions:**

Our study provides the single-cell transcriptional landscape of in vitro DA differentiation, which can guide future improvements in DA preparation and quality control for PD cell therapy.

**Supplementary Information:**

The online version contains supplementary material available at 10.1186/s13287-022-03104-7.

## Introduction

Parkinson's disease (PD) is a common neurodegenerative disease [1, 2], which is pathologically characterized by the degeneration of midbrain dopaminergic (DA) neurons in the substantia nigra [3–5]. The rapid aging of populations makes PD a growing social burden in the world [6]. Due to the limited repair capacity of the adult central nervous system, missing nerve cells are unable to regenerate. Several clinical studies suggested that cell therapy with DA neurons from human fetal tissue can ameliorate the symptoms of PD [4, 7–12]. Most importantly, some patients obtained very significant and long-term benefits [13, 14], indicating the great potential of cell-based therapy in the treatment of PD. However, the ethical concerns and standardization challenges hampered the clinical application of fetal tissues [15]. Human pluripotent stem cells (hPSCs) derived DA cells are a promising substitute for cell transplantation [16, 17]. During the last decade, numerous protocols for DA neurons differentiation from hPSCs have been developed by several groups [18–22], and the cell transplantation of hPSC-derived DA cells has been used with success in animal models [23–25]. Recently, a clinical trial also exhibited the feasibility of autologous iPSC-derived DA cell transplants [26]. However, there is still a challenge to selectively obtain pure DA progenitors or neurons from hPSCs [15, 27]. Most studies suggest that the percentage of TH^+^ cells in the graft is 10–30% [23, 24, 28], single-cell level analysis reveals several unexpected cell types, such as astrocyte, vascular leptomeningeal cell (VLMC) [29, 30] and non-DA neurons [31], which may influence the in vivo evaluation of DA cells and even increase the risk of side effects [27]. All those findings demonstrated that the regulation of DA cell fate is currently not well characterized, and more efficient and reliable differentiation or purification strategies are urgently needed.

In this study, we used scRNA-seq to transcriptionally profile the in vitro DA differentiation at different stages from hESCs. We aimed to provide a roadmap of hESC-derived DA progenitors to accurately understand the identity of cell derivations. Our results identified three branch points by lineage trajectory analysis. We found several genes might involve in DA fate choice and validated their functions through the genetic gain- and loss-of-function approaches. Further analysis indicated that choroid plexus epithelial cells (CPECs) are the main non-DA cell type, and CD99 is a candidate surface marker for the purification of DA progenitors. Our findings provide a valuable strategy for enhancing DA in vitro differentiation from hPSCs.

## Materials and methods

### hESCs culture and DA differentiation

hESCs line Q-CTS-hESC-2 was maintained in Essential 8™ Medium (E8, Gibco, Grand Island, NY, USA) on vitronectin (Gibco)-coated dishes with medium changed every day. Cells were passaged by CTS™ TrypLE™ solution every 4–5 days. For DA differentiation, a previously described protocol [20, 21] was used with modifications. Briefly, Q-CTS-hESC-2 was dissociated into single cells and plated on vitronectin-coated dishes at a density of 2 × 10^4^ cells/cm^2^ in E8 medium containing 10 µM Y-27632 (Selleck, Houston, Texas, USA). From day 0 to 8, the medium was changed to N2 induction medium (CTS™ KnockOut™ DMEM/F-12 (Gibco), CTS™ Neurobasal® Medium (Gibco), 1% (v/v) N2 Supplement (Gibco), 2 mM CTS- GlutaMAX™-I (Gibco), supplemented with 100 nM LDN193189 (Stemgent, Boston, Massachusetts, USA), 10 µM SB431542 (Stemgent), 100 ng/mL SHH (R&D), 2 μM SAG (Calbiochem, Darmstadt, Germany) and 0.7 µM CHIR99021 (Stemgent), and the medium was changed every 2 days. On day 9, the N2 induction medium was supplemented with 100 ng/mL SHH, 0.7 µM CHIR99021, and 100 ng/mL FGF8 (Peprotech, Cranbury, NJ, USA). On day 11, we performed cell passaging and the medium was changed to B27 medium (CTS™ Neurobasal® Medium, 2% (v/v) B27 Supplement (Gibco), 2 mM CTS-GlutaMAX™-I) supplemented with 20 ng/mL (Peprotech), 20 ng/mL GDNF (Peprotech), 0.2 mM Ascorbic acid (Sigma, St Louis, MO, USA), 100 ng/mL FGF8 and 1 ng/mL TGFβ3 (Peprotech). From day 11 to 16, the medium was same, and the medium was changed every 2 days. On day 17, the cells were passaged secondly, and medium was supplemented with 20 ng/mL BDNF, 20 ng/mL GDNF, 0.2 mM Ascorbic acid AA, 1 ng/mL TGFβ3, 500 μM cAMP (Sigma), and 10 μM DAPT (Tocris, Bristol, UK).

### Immunocytochemistry and microscopy

Cells were pre-seeded on the coverslips and fixed in 4% PFA for 30 min at room temperature (RT), then washed three times with PBS. For staining, the cells were blocked with TBS (1 × PBS + 0.3% Triton + 2% bovine serum albumin) for 1–2 h before adding the primary antibodies solution, incubated with primary antibody overnight at 4 °C, and rinsed three times in PBS before adding secondary antibody. Cultures were incubated with secondary antibodies for 2 h and finally washed three times. The nuclei were visualized using hoechst33342 for 10 min. All images were captured by Zeiss confocal microscope LSM 780/880.

### Antibodies

Primary antibodies included rabbit anti-OCT4 (Santa Cruz; sc-9081; 1:200), goat anti-FOXA2 (R&D; AF2400; 1:500), rabbit anti-LMX1A (Millipore; ab10533; 1:1000), mouse anti-EN1 (DHSB; 4G11; 1:50), mouse anti-NURR1 (R&D; pp-N1404; 1:500), rabbit anti-TH (Millipore; ab152; 1:1000), mouse anti-TH (Immunostar; 22941; 1:2000), mouse anti-CD99 FITC (BioLegend; 371303; 1:50).

Secondary antibodies were all used at 1:200 dilution: anti-rabbit cy3 (Jackson Laboratories; 711-165-152), anti-rabbit cy5 (Jackson Laboratories; 711-605-152), anti-mouse cy3 (Jackson Laboratories; 715-165-151), anti-mouse cy5 (Jackson Laboratories; 715-605-151), anti-goat cy3 (Jackson Laboratories; 705-165-147), anti-goat cy5 (Jackson Laboratories; 705-605-147).

### Cell sorting

Day 25 cells were dissociated into single-cell suspension with CTS™ TrypLE™ and filtered using a 40-μm mesh filter. Cells were counted and resuspended at a density of 1 × 10^7^ cells/mL. Cells were stained at a 37 °C incubator for 20 min using a 1:100 dilution of a FITC-conjugated anti-CD99 antibody and an Alexa 488-conjugated anti-mouse IgG2a antibody. Then, the cells were washed twice with 15 mL of cell medium and resuspending to 1 × 10^7^ cells/mL for sorting. According to the operating steps, use the BD LSRFortessa™ X-20 cell analyzer to sort CD99 negative and CD99 positive cells for further culture and identification. The sorted cells were collected and replanted in 4-well plates with a culture medium containing 10 µM Y-27632. The sorting process must be strictly operated in accordance with the aseptic technique.

### Single-cell isolation

At each differentiated stage (day 0, 5, 11, 17, and 25), differentiated cells were dissociated into single cells with CTS™ TrypLE™ and neutralized with corresponding mediums. Single-cell suspensions were filtered through a 40-μm mesh filter. Cells were centrifuged at 1200 rpm for 3 min and resuspended in PBS containing 0.04% BSA to a concentration of 1 × 10^6^ cells/mL. Approximately 10,000 cells were loaded onto a 10x (10 × Genomics Chromium Single-Cell System) chip for a target recovery of 6000 cells.

### scRNA-Seq analysis

We used single-cell 3 'Library and Gel Bead Kit V3 (10 × Genomics, 1,000,075, http://www.10xgenomics.com/) and Chromium Single-Cell B Chip Kit (10 × Genomics, 1,000,074) for cell capture, cDNA synthesis, and libraries according to the manufacturer’s instructions. Libraries were sequenced on Illumina Novaseq6000 sequencer with a sequencing depth of at least 100,000 reads per cell with pair-end 150 bp (PE150) reading strategy. Sequencing data were first preprocessed through the Cell Ranger pipeline (10 × Genomics, Cellranger count v5) with default parameters (expect cells set to the number of cells added to 10 × system), aligned to GrCH38 (v3.0.0) and resulting matrix files were used for subsequent bioinformatics analysis [32]. Seurat (version 3.1.5 and R version 4.0.2, R) was utilized for downstream analysis [33]. Batch effects were removed using the Harmony algorithm (1.0), treating individual 10 × runs as a batch [34]. Cells with at least 200 detected genes were retained, and the data were normalized to transcript copies per 10,000 and log-normalized to reduce sequencing depth variability. For visualization and clustering, manifolds were calculated using UMAP methods [35] (RunUMAP, Seurat) on 20 precomputed principal components. Clusters were identified by calculating a shared-neighbor graph and then defined (Find-Clusters, Seurat) with a resolution of 0.2. Identification of differentially expressed genes between clusters was carried out using the default Wilcoxon rank sum test (Seurat). For pseudo-time and trajectory analysis Monocle 3 [36, 37] (1.4) was adopted.

### qPCR

RNA was extracted using TRIzol™ (Invitrogen, 15,596,018). Hifair™ II 1st Strand cDNA Synthesis SuperMix Kit (Yeasen, 11123ES60) was used for cDNA synthesis. Quantitative real-time PCR was performed with Hieff UNICON™ Universal Blue qPCR SYBR Green Master Mix (Yeasen, 11184ES08) according to the manufacturer’s protocol. Gene expression levels were normalized to *GAPDH*. Primers for real-time qPCR are listed in Additional file1: Table S1.

## Results

### Single-cell RNA sequencing analysis of DA differentiation

**Fig. 1:**
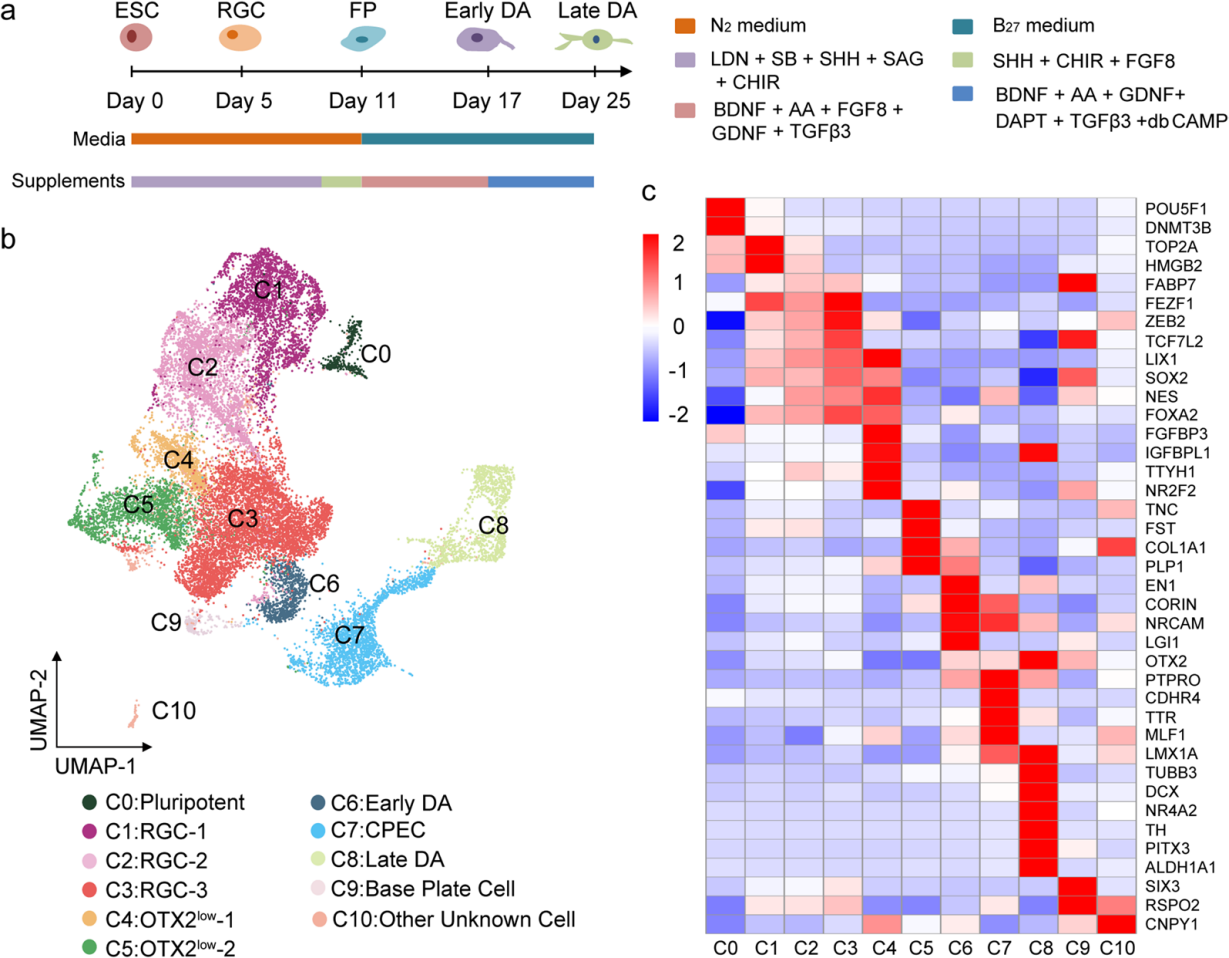
Single-cell RNA sequencing of DA differentiation in vitro*. a* Schematic depicting DA differentiation from hESCs at different differentiation stages. **b** We defined 11 clusters in DA differentiation. **c** Heat map showing expression levels of the differentially expressed genes of each cluster

To determine the genetic regulation of DA differentiation, we used a widely adopted floor plate-based protocol [20, 21] with modifications, which produces a high percentage of cells positive for traditional floor plate markers (FOXA2^+^*/*LMX1A^+^) from hPSCs. The differentiating cells from ESCs to DA progenitors at five time points (day 0, day 5, day 11, day 17, and day 25), which spanned radial glial (RG) cells (day 5), early floor plate (day 11), early DA progenitors (day 17) and late DA progenitors (day 25) were collected for single-cell transcriptional analysis (Fig. 1a). A total of 21,981 cells were captured, and 19,714 cells passed the quality tests. We used Seurat for downstream analysis and Harmony to eliminate batch effects. Cells derived from five differentiation stages were visualized on a uniform manifold approximation and projection (UMAP). Based on known feature genes expression, we identified 10 clusters of differentiated cells: three types of radial glial cells (RGCs) (C1-C3, expressing *FABP7, FEZF1, SOX2, HMGB2, and TCF7L2*), two types of DA progenitors (C6, expressing floor plate markers *LMX1A, FOXA2, CORIN, OTX2,* and the midbrain marker *EN1*; C8, expressing neuron markers *TUBB3B*, *DCX* and DA markers *NR4A2*, *TH*, and *PITX3*), and five types of non-DA cells (C4 and C5, *OTX2*^*low*^ cells, weakly expressing *OTX2*; C7, CPECs, expressing *TTR* and *PTPRO*; C9, base plate cells, expressing *SOX2, HMGB2*, and *FOXA2*; C10, other unknown cells) (Fig. 1b, c). GO terms analysis (GO) showed that the expression levels of cycling genes and cell proliferative markers *TOP2A* and *MKI67* gradually decreased from C1 to C3 (Fig. 1c and Additional file2: Fig. S1a, b), and this is also consistent with recently published article [30].

### Trajectory mapping reveals three branch points in DA differentiation

**Fig. 2:**
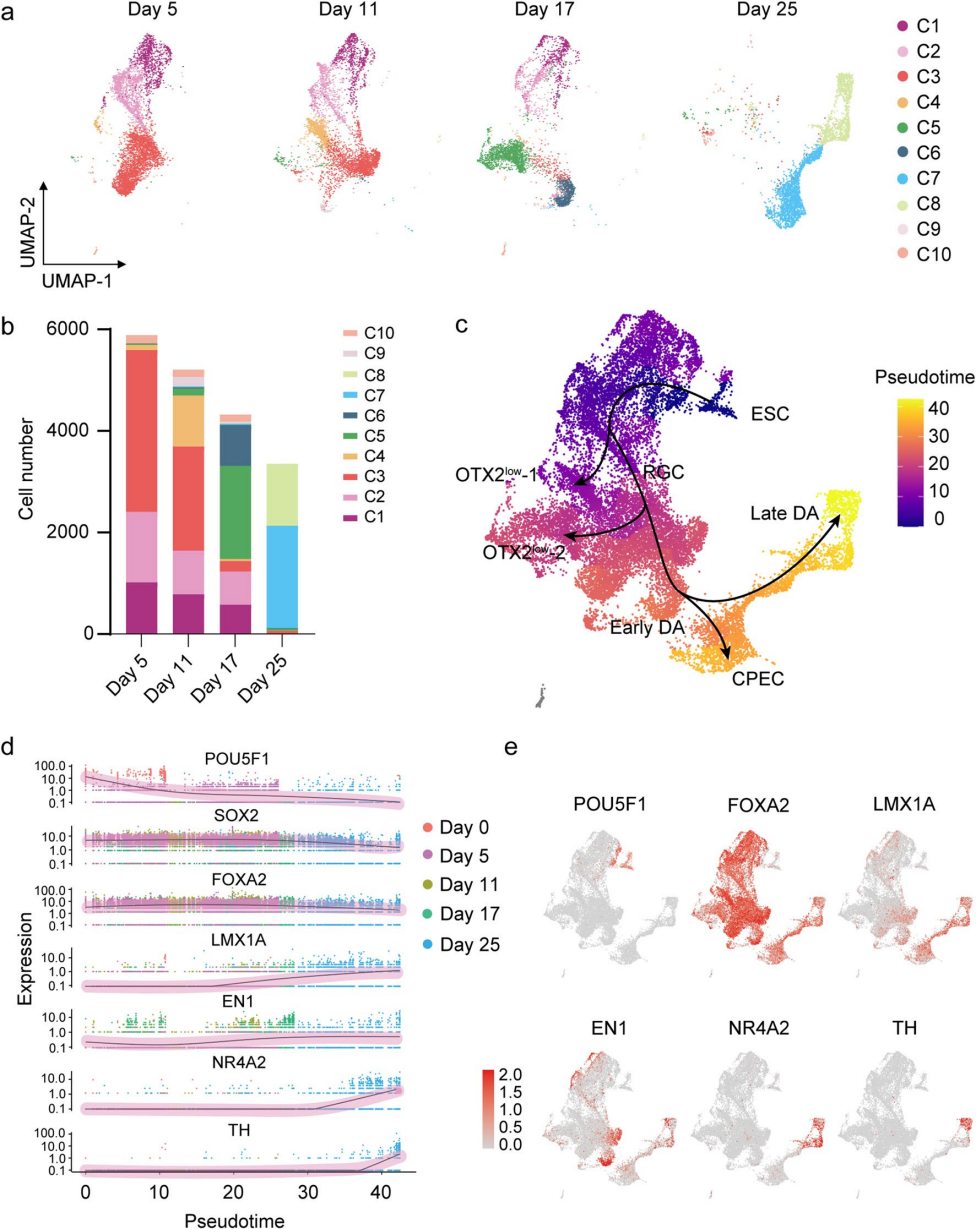
Differentiation trajectory from hESCs to DA. **a** UMAP projections of cells sampled from day 5 to 25. **b** Cell numbers at each time point. **c** Pseudo-time trajectories calculated from UMAP **d** Expression of selected marker genes along pseudo-time. **e** Expression of marker genes projected onto UMAP

UMAP projections of cells from day 5 to 25 revealed dynamic changes in cell populations over time (Fig. 2a). On day 5, hESCs first give rise to RGCs, accompanied by downregulation of pluripotent genes *POU5F1* and *DNMT3B* (Fig. 1c). We found that RGCs were the prominent clusters at early stages. Day 5 and day 11 cells contained 95% and 70% of RGCs, respectively (Fig. 2a, b). Early DA progenitors (C6) appeared on day 17, while young DA neurons (C8) emerged on day 25. In contrast, the two *OTX2*^*low*^ non-DA populations were detected on day 11 (C4, 20% of cells) and day 17 (C5, 42% of cells), respectively. Notably, we also identified a CPECs population (C7) specific to day 25. Moreover, two rare cell types (< 5% of cells at any time point), including base plate cell population (C9) and an unknown population (C10), were detected in the differentiation process.

We next investigate the lineage trajectories from hESCs to DA progenitors using Monocle 3. Pseudo-time sequence analysis revealed that the DA lineage (C1: C2: C3: C6: C8) diverts from hESCs into RGCs populations (C1, C2, C3) on day 5 and turns into the early DA progenitors (C6) on day 17, which further mature into young DA neurons (C8) (Fig. 2c). The trajectory mapping revealed three branching points from pluripotent cells. The first branch gives rise to an *OTX2*^*low*^ population (C2: C4) on day 11, indicating part of the RGCs differentiate to a fate of non-DA lineage. The second branch generates from RGCs into another *OTX2*^*low*^ population (C3: C5) on day 17. The third branch gives rise to CPECs (C6: C7) on day 25. Expression of known markers during DA differentiation displaying expected temporal-specific transcriptional profiles (Fig. 2d, e). Specifically, the expression of pluripotency gene *POU5F1* (*OCT4*) is drastically reduced from day 0 to 5, whereas the ventral midbrain marker *FOXA2* is highly expressed from day 5 and sustained high expression to day 25. The midbrain marker *EN1* reached a peak on day 17, which at the key branching point between C5 and C6, and DA marker *NR4A2* and *TH* increased between day 17 and 25. These results are confirmed by protein level analysis using immunofluorescent staining (Additional file2: Fig. S2a, b). Our findings established a continuous trajectory map of DA lineage differentiation in vitro. In particular, *EN1* was found to be a key marker specific for DA progenitors, consistent with previous work showing that *EN1* is essential for the repression of undesired cell types [18]. Moreover, we observed genes that largely coincide with the expression of *EN1*, which may play a role in DA differentiation.

### Molecular regulation of the key lineage branch point on day 17

**Fig. 3:**
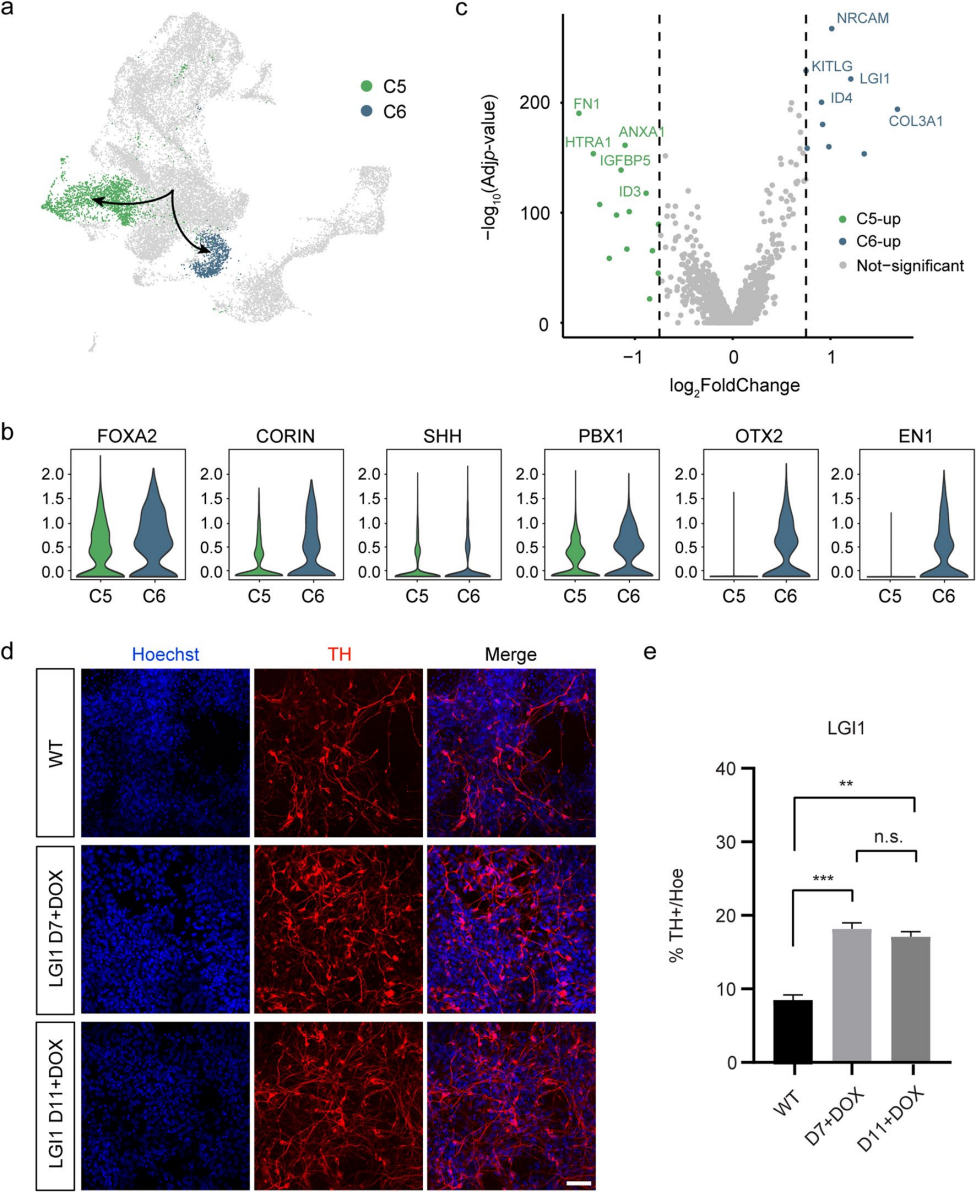
Defining two molecularly distinct populations in the key branch point on day 17. **a** The branch point between day 11 and 17 during DA differentiation. **b** C5 and C6 clusters showing expression of DA progenitors-related genes shown as violin plots. Violin plots showing the expression of marker genes of C5 and C6 clusters. **c** Volcano plot of significantly differentially expressed genes (|log_2_FC|> 1; *p*_adj_ < 0.05) between C5 and C6 cluster. **d** Immunofluorescence detection of TH positive after the hESCs cell lines of overexpressing *LGI1* differentiation into DA. n.s., not significant. Data are shown as mean ± SEM. *P < 0.05, **P < 0.01, ***P < 0.001. Scale bars, 50 μm. **e** Quantification of the percentage of TH^+^ cells on day 25 of differentiation. Dox was added on day 7 and day 11, respectively. Data are shown as mean ± SEM, *n* = 3. **P < 0.01, ***P < 0.001

Trajectory analysis revealed the key branch point between day 11 and 17, which gave rise to C5 and C6 populations (Fig. 3a). These clusters both expressed floor plate markers *FOXA2, SHH, PBX1*, and *CORIN*; however, C5 differed mainly from C6 clusters in its lower or no expression of *OTX2* and *EN1* (Fig. 3b). A comparison of differentially expressed genes (DEGs) revealed gene expression profiles between the two populations. We found a total of 322 differentially expressed genes and demonstrated their expression at the single cell level by cluster-based heatmap. Notably, C6 robustly and selectively expresses *NRCAM, KITLG, LGI1*, and *ID4*, whereas *FN1, ANXA1, HTRA1*, *IGFBP5*, and *ID3* were confined to only C5. (Fig. 3c). Based on the enriched genes in C6, we next aimed to investigate whether these genes could promote DA cell specification. Using the CRISPR/Cas9 system, we established three doxycycline (dox)-inducible knockdown cell lines, including *ID3*, *GSC*, and *FEFF2*, and three dox-inducible gene overexpression lines, including *ID4*, *LGI1*, and *NR6A1. LGI1* (leucine-rich glioma inactivated 1), a neuronal secreted protein that has previously been shown to be an essential player in nervous system development [38, 39] and neuronal growth [40]. Several studies have also shown that patients with *LGI1* autoantibody disease exhibited Parkinsonism symptoms [41, 42]. Immunofluorescence analysis revealed that inducing overexpression of *LGI1* on both day 7 and day 11 significantly increases the proportion of TH^+^ cells (*P* < 0.001; Fig. 3d, e). This is consistent with the qPCR data. Notably, overexpression of *LGI1* on day 7 significantly enhanced the expression of *EN1*, *OTX2*, and *NR4A2* (Additional file2: Fig. S3). We also confirmed the efficient upregulation of *LGI1* mRNA (16.5-fold above WT levels) after dox induction for 3 days by RT-qPCR. Our results indicate that *LGI1* contributes to inducing DA progenitor fate.

### The subpopulations analysis of cells on day 25

**Fig. 4:**
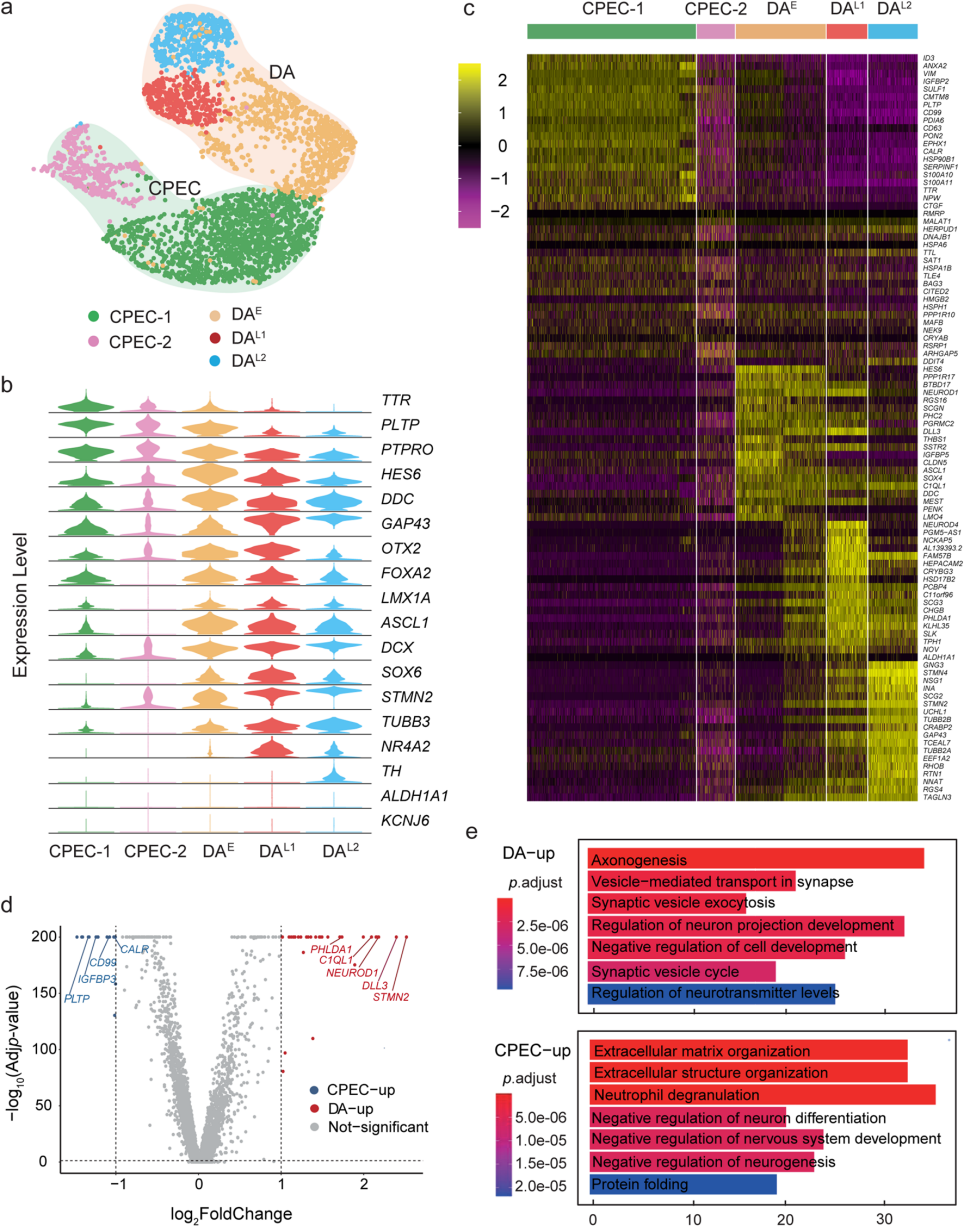
Subpopulations and their molecular signature of day 25 cells. **a** UMAP map of the five subpopulations of day 25 cells. Cell-type assignments are indicated. **b** Violin plots showing expression of key lineage-associated genes between the five subpopulations. **c** Heatmap showing expression levels of DEGs for each subcluster. **d** Volcano plot of significantly DEGs (|log_2_FC|> 1; *p*_adj_ < 0.05) identified by choroid plexus cells versus DA progenitor cells. **e** GO analysis of differentially expressed genes governing DA and CPECs

Postmitotic DA progenitors around day 25 of differentiation are widely used for transplantation in animal PD models [19, 20, 24, 43] and patients [26]. In our protocol, it is an important process in cell fate selection from day 17 to 25, which mainly gave rise to C7 and C8. To clarify the composition and cell type-specific transcriptional signatures of day 25 cell products, we performed unsupervised analysis or reran the integration (Harmony) and clustering of day 25 cells and identified five subpopulations. CPEC-1 was *TTR*^+^, *PLTP*^+^, *PTPRO*^+^, *FOXA2*^+^ and *LMX1A*^+^, and CPEC-2 was *TTR*^+^, *PLTP*^+^, *PTPRO*^+^, *FOXA2*^−^ and *LMX1A*^−^. The neuronal clusters shared the expression of neuronal precursor markers *ASCL1*, *STMN2*, and *DCX*, DA markers *LMX1A*, *FOXA2*, *OTX2*, and *DDC*, suggesting a DA precursor fate. Whereas using genes that reflect early DA (*DCX*^+^/*DDC*^+^/*NR4A2*^−^) versus late stages of DA progenitor (*DDC*^+^/*NR4A2*^+^/*TH*^+^), these cells were defined as early DA precursor (DA^E^) and late DA precursor (DA^L1^, DA^L2^) (Fig. 4a, b), indicating a maturation gradient of cells. Previous work showed that forced expression of SOX6 in human DA progenitors strongly induces A9-like DA subtype [44]. We noted that all the three DA populations expressed *SOX6*, raising the possibility for A9 cell generation. By contrast, *KCNJ6* or *ALDH1A1* was not detected at this stage, indicating a need for prolonged maturation culture for DA subtype specification (Fig. 4b). DEG analysis revealed distinct transcriptional properties of these subpopulations and further supported this annotation (Fig. 4c). Next, we compared the molecular regulations between CPECs and DA populations and found 817 differentiation genes. Some of the topmost variable genes that identify C7 and C8 appear to distinguish the two populations (Fig. 4d), which may contribute to the purification of the final product. GO term enrichment analysis of these two cell types revealed that genes associated with the biological processes of axonogenesis and synapse function were enriched in DA precursors. In contrast, CPECs showed enrichment of extracellular matrix-related genes and negative regulation of neural genes (Fig. 4e).

### Enrichment of DA progenitors by CD99 sorting

**Fig. 5:**
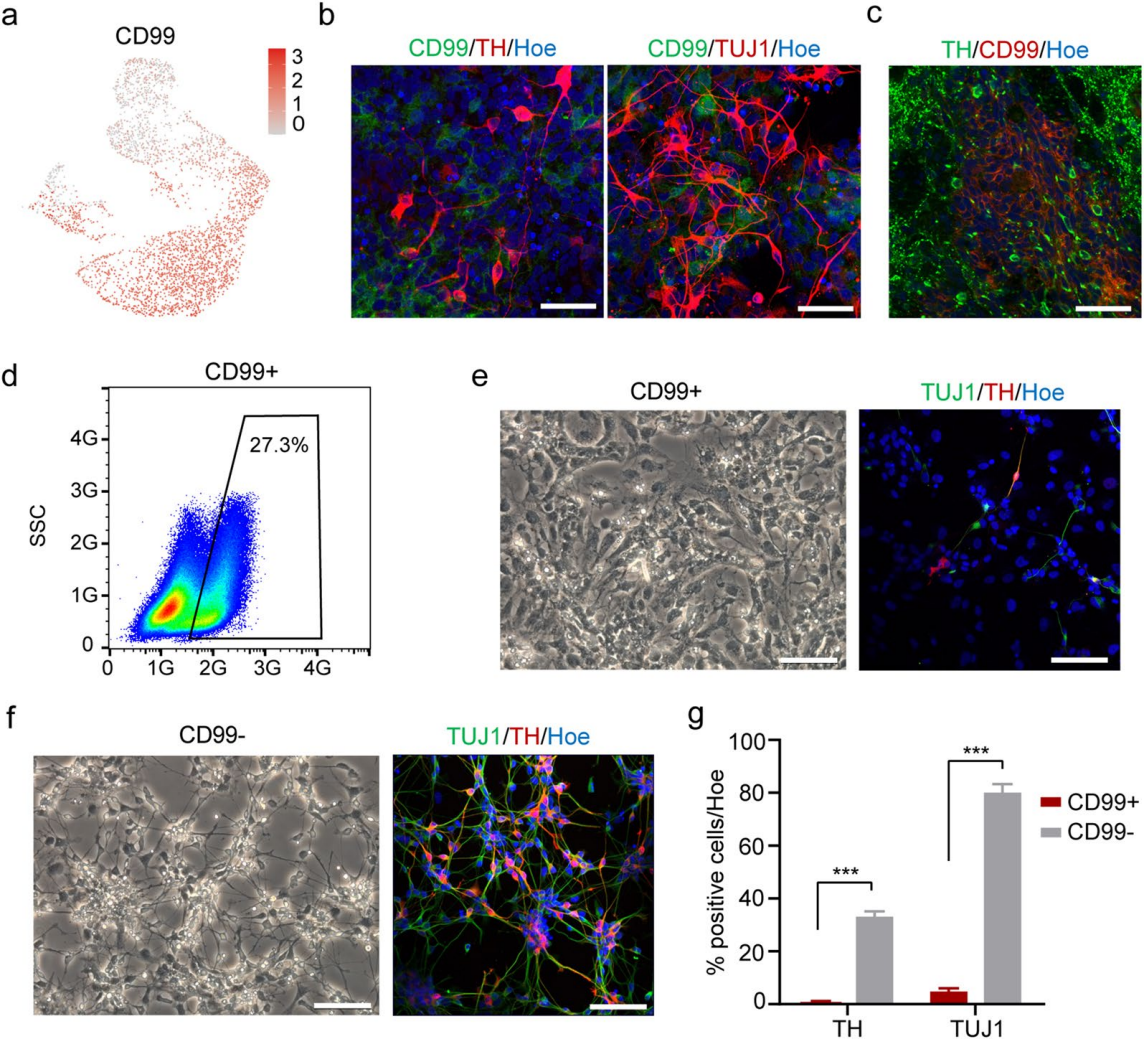
Enrichment of DA progenitors with CD99. **a** CD99 expression in day 25 cells. **b**, **c** Immunofluorescence for CD99 of day 25 cells (**b**) and of grafts derived from rat striatum at 3 months after transplantation (**c**). Scale bars, 50 μm. d FACS analysis of CD99^+^ cells in day 25 cells. **e**, **f** Cell morphology and immunofluorescence for CD99^+^ cells (**e**) and CD99^−^ cells (**f**). Scale bars, 50 μm. **g** The percentages of TUJ1^+^ and TH.^+^ cells in sorted cells. Data are shown as mean ± SEM, *n* = 3 independent experiments. ***P < 0.001

Given the DA progenitor and contaminating populations of day 25 product have been defined, a key next step is to explore ways of specifically enriching DA progenitors. Our analysis identifies *CD99* as a potential surface marker candidate, which is specifically expressed in CPECs (Figs. 4d, 5a). To assess whether the two populations can be distinguished by CD99, we performed immunofluorescence staining of day 25 cells. The results indicate that CD99^+^ cells exhibit non-neuronal morphology and do not express TH or TUJ1 (Fig. 5b). We also found CD99^+^ cells in the cell grafts in vivo (Fig. 5c). A flow cytometric analysis revealed that the percentage of CD99^+^ cells was about 27.3% on day 25 (Fig. 5d). Moreover, CD99^+^ and CD99^−^ cells were collected by fluorescence-activated cell sorting (FACS) on day 25 and were cultured overnight. Double-labeled immunostaining revealed that there were very few of the CD99^+^ cells expressing TUJ1 (5.57% ± 0.77) or TH (1.16% ± 0.05). However, most CD99^−^ cells were positive with TUJ1 (80.79% ± 4.28) and TH (33.91% ± 2.05) (Fig. 5e–g). Cell morphology of sorted cells further supported the accuracy of our purification strategy. Together, our data revealed that CD99 could be used as a cell surface marker for enriching DA progenitors.

## Discussion

PD cell therapy has long been a front runner in the cell replacement study. Nonetheless, undesired populations are produced in the cell differentiation process and can hardly be defined by the current markers. In this study, we perform single-cell RNA sequencing (scRNA-seq) to characterize the cells formed during DA differentiation.

We analyzed nearly 20,000 cells at different time points, which spanned the transition from pluripotency stage to post-miotic DA progenitors. We find that the directed differentiation gives rise to several cell populations at different stages. Unexpectedly, cells on day 5 begin to exhibit heterogeneity with a small amount of aberrant cell types. PSCs first generate radial glia cells with different proliferation ability and floor plate features, consistent with previous work [45, 46]. Radial glia cells are the dominant cell types at early time points and subsequently generate DA progenitors in a temporal pattern as well as non-DA contaminants. Previous studies have indicated that non-DA cells are generated in mid- to late-stage (around day 30) of differentiation [46, 47], or stage of terminal differentiation into DA neurons [30, 46]. Our analysis of composition over time revealed that non-DA populations started to emerge in the differentiation from radial glia cell to early floor plate (days 5–11), highlighting the importance of identifying early fate determinants.

We identified several feature genes of DA progenitors at lineage branch points, which are potentially involved in DA differentiation. *LGI1*, for example, was found highly expressed in EN1^+^ DA progenitor clusters. Our study indicated that forced expression of *LGI1* at an early stage can partially induce cell fate transition toward DA identities, suggesting its potential role in DA commitment. Importantly, we identify CD99 as a surface marker which could be used to eliminate CPECs and improve the purity of DA progenitors via FACS isolation. Additionally, no AQP4^+^ astrocytes or 5-hydroxytryptamine (5-HT^+^) serotonergic neurons are detected in the final cell product, which has been reported in some of the in vivo grafts [25, 30]. In conclusion, our findings provide valuable resources for identifying gene expression patterns associated with DA patterning in vitro and can be utilized to guide human DA differentiation and assessment.

## Conclusions

Overall, we have conducted a comprehensive and detailed analysis of DA cells derived from hESCs and revealed DA differentiation trajectory, as well as three lineage branching points. We described distinct gene expression profiles of major cell populations and identify CD99 as a surface marker for the elimination of major contaminant cells in the final cell product, which will help to improve the in vitro differentiation protocol and the cell preparation used for cell therapy.

## Supplementary Information

**Additional file1.** **Table S1**: List of primers for real-time PCR.

**Additional file2**. **Figure S1**: Cell proliferation assay in UMAP. a Expression of cell proliferative markers TOP2A and MKI67. b UMAP projection of predicted cell cycle phases. **Figure S2**: Characterization of hESC-derived DA cells at different time points. a Immunofluorescence staining of pluripotency gene (OCT4) and floor plate markers (FOXA2 and LMX1A). b Immunofluorescence staining of midbrain marker (EN1) and DA markers (NURR1 and TH). Scale bars, 50 μm. **Figure S3**: qPCR analysis of LGI1 overexpression cells on day 25 of DA differentiation. Dox was added on day 7 and day 11, respectively. Data are shown as mean ± SEM, n = 3. *p < 0.05, **p < 0.01, ***P < 0.001.

## Data Availability

The datasets used and/or analyzed during the current study are available from the corresponding author on reasonable request.

## Abbreviations

DA: Dopaminergic
hPSCs: Human pluripotent stem cells
PD: Parkinson’s disease
hESCs: Human embryonic stem cells
scRNA-seq: Single-cell RNA sequencing
VLMC: Vascular leptomeningeal cell
RGCs: Radial glial cells
GO: Gene ontology
CPECs: Choroid plexus epithelial cells
FACS: Fluorescence-activated cell sorting
UMAP: Uniform manifold approximation and projection
