# Supplementary material for: Single-cell transcriptomics reveals the cell fate transitions of human dopaminergic progenitors derived from hESCs
Source: Stem Cell Res Ther. 2022 Aug 13;13:412. doi: 10.1186/s13287-022-03104-7 (PMC9375405; doi:10.1186/s13287-022-03104-7)
Supplement: Supplementary file 2 — Additional file2. Figure S1: Cell proliferation assay in UMAP. a Expression of cell proliferative markers TOP2A and MKI67. b UMAP projection of predicted cell cycle phases. Figure S2: Characterization of hESC-derived DA cells at different time points. a Immunofluorescence staining of pluripotency gene (OCT4) and floor plate markers (FOXA2 and LMX1A). b Immunofluorescence staining of midbrain marker (EN1) and DA markers (NURR1 and TH). Scale bars, 50 μm. Figure S3: qPCR analysis of LGI1 overexpression cells on day 25 of DA differentiation. Dox was added on day 7 and day 11, respectively. Data are shown as mean ± SEM, n = 3. *p < 0.05, **p < 0.01, ***P < 0.001. [file 13287_2022_3104_MOESM2_ESM.docx]

**
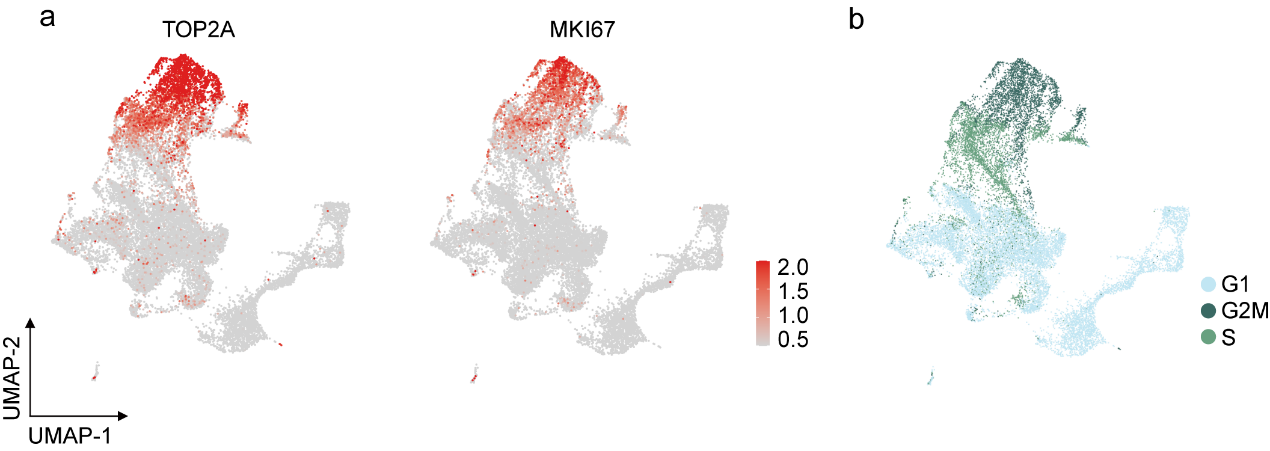
Fig.S1** Cell proliferation assay in UMAP*.* **a** Expression of cell proliferative markers *TOP2A* and *MKI67*. **b** UMAP projection of predicted cell cycle phases.


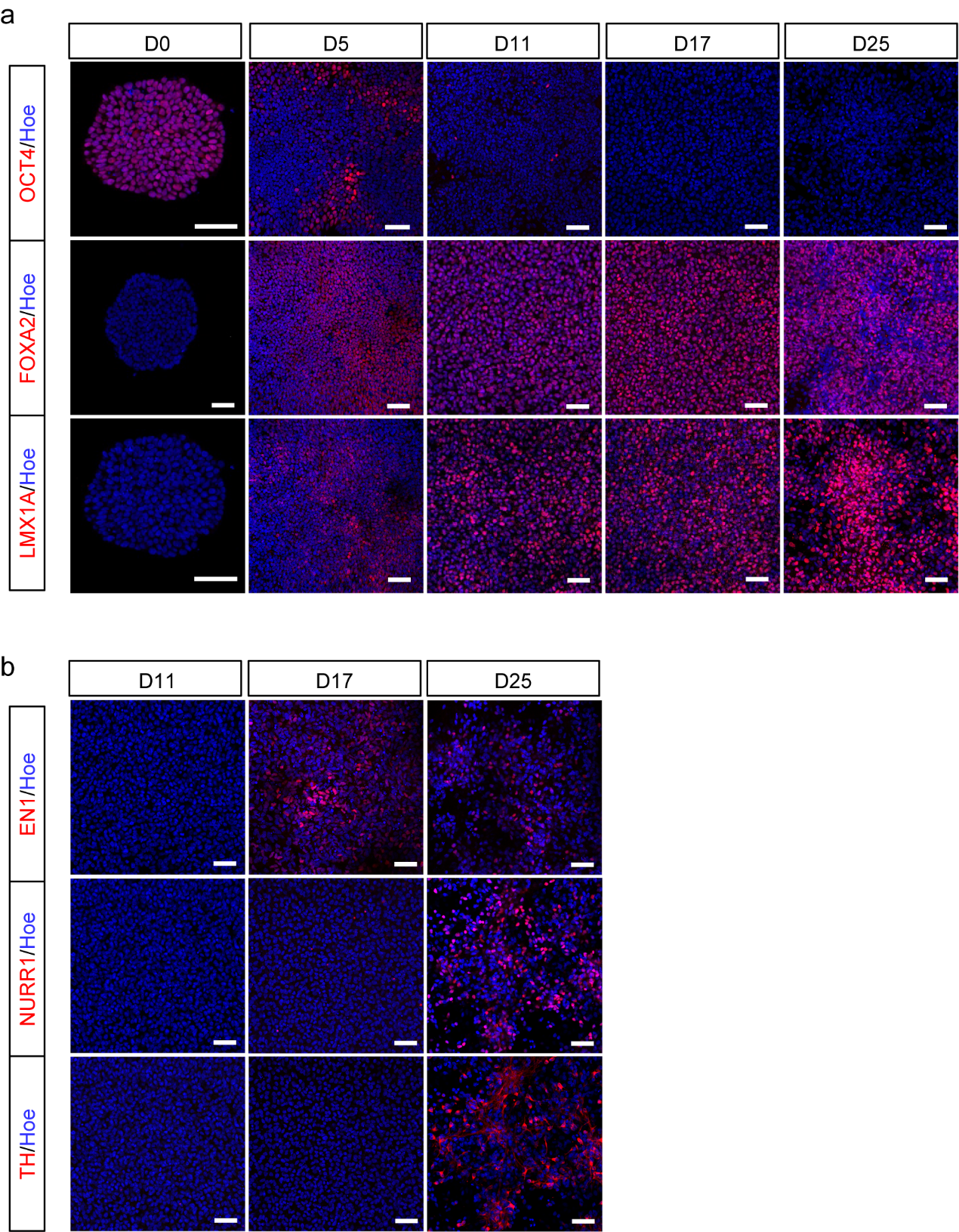


**Fig.S2** Characterization of hESC-derived DA cells at different time points. **a** Immunofluorescence staining of pluripotency gene (OCT4) and floor plate markers (FOXA2 and LMX1A). **b** Immunofluorescence staining of midbrain marker (EN1) and DA markers (NURR1 and TH). Scale bars, 50 μm.


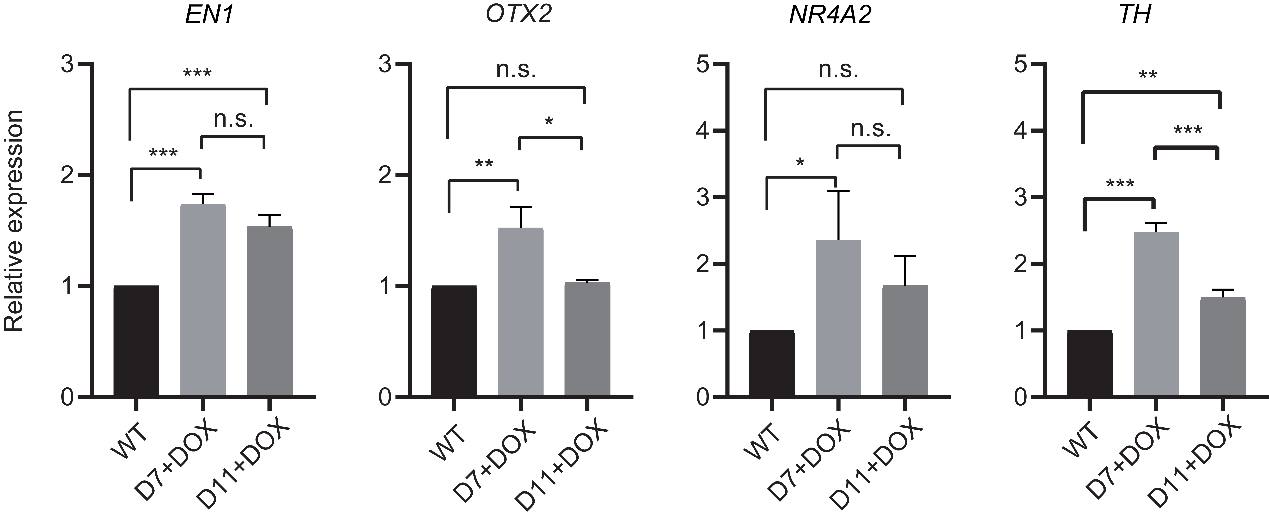


**Fig.S3** qPCR analysis of *LGI1* overexpression cells at day 25 of DA differentiation. Dox was added on day 7 and day 11, respectively. Data are shown as mean ± SEM, n = 3. *p < 0.05, **p < 0.01, ***P < 0.001.
